# Editorial

**Published:** 1912-03

**Authors:** 


### BUFFALO MEDICAL JOURNAL

**A Monthly Review of Medicine and Surgery**

*EDITOR*

A. L. BENEDICT.

All communications, whether of a literary or business nature, books for review and exchanges, should be addressed to the editor, 354 FRANKLIN STREET, BUFFALO, N. Y Please make personal or telephonic calls before 1 p. m

THE BUFFALO MEDICAL JOURNAL will publish regularly. full transactions of any professional organization in western New York at cost. Personal notices, brief reports of society proceedings, and the like, are always welcome and will be published without charge.

Subscriptions may commence at any time. $2.00 per year in advance,

VOL. LXVII. MARCH, 1912 No. 8

"LEST WE FORGET." The editor receives many kind words of congratulation on the growing usefulness of the BUFFALO MEDICAL JOURNAL. He will try to merit these compliments and, indeed, is incurring the risk of an intestinal diverticulum from the effort put forth. But, let us not forget the very loyal assistance, in many ways, of named and unnamed collaborators. And let us not forget the gallant veteran who spent so many years of his maturity in the task begun when he was older than the present editor and who kept up the work at an age and under stress of physical suffering, which would drive most of the younger generation into retirement. Col. Potter directed the JOURNAL even from his death bed, with skilled and loyal assistance, it is true, but putting his own personal seal upon the last number, issued during his life time, a year ago. One of the most touching episodes in his long and varied career, was the almost paternal eagerness with which he took into his hands, the March 1911 copy, fresh from the press, knowing that it would be the last of his literary offspring and having, indeed, scarcely hoped to survive till it should arrive. We are fortunate in being able to present, for several issues to come, his memoirs of the Civil War which he had prepared for posthumous publication and whose value will be as high in the distant future, as at present, when everything else in the volume will have been superceded by technical knowledge and professional issues which we cannot even imagine.

